# Association between HLA-DP Gene Polymorphisms and Cervical Cancer Risk: A Meta-Analysis

**DOI:** 10.1155/2018/7301595

**Published:** 2018-06-13

**Authors:** Lin Cheng, Yan Guo, Shipeng Zhan, Peiyuan Xia

**Affiliations:** ^1^Institute of Burn Research, The First Affiliated Hospital of Third Military Medical University (Army Medical University), Chongqing 400038, China; ^2^Department of Pharmacy, The First Affiliated Hospital of Third Military Medical University (Army Medical University), Chongqing 400038, China; ^3^Department of Infectious Disease, The First Affiliated Hospital of Third Military Medical University (Army Medical University), Chongqing 400038, China

## Abstract

*Objective. *We aimed to derive a more precise estimation of the associations between human leukocyte antigens DP (HLA-DP) gene polymorphisms and cervical cancer risk by meta-analysis.* Methods. *PubMed, EMBASE, ScienceDirect, Web of Science, Chinese National Knowledge Infrastructure (CNKI), and Wanfang databases were systematically searched to identify studies investigating the relationship between HLA-DP gene polymorphisms and cervical cancer. The associations between them were evaluated by pooled OR and 95% CI.* Results. *A total of 11 studies including 5008 cases and 9322 controls with 11 HLA-DP alleles were included in the current meta-analysis.* Results. *The results showed that HLA-DPB1⁎03:01 was significantly associated with an increased risk of cervical cancer (OR=1.252, 95%CI: 1.116-1.403, Pz=0.001), while HLA-DPB1⁎04:02 and HLA-DP rs3117027 G allele were significantly associated with a decreased risk of cervical cancer (OR=0.744, 95%CI: 0.652-0.848, Pz=0.001; OR=0.790, 95%CI: 0.745-0.837, Pz=0.001), and HLA-DP rs9277535 G allele was significantly associated with a decreased risk of cervical cancer in Asia (OR=0.802, 95%CI: 0.753-0.855, Pz=0.001). Subgroup analyses based on race system showed that HLA-DPB1⁎13:01 was significantly associated with an increased risk of cervical cancer in Asia (OR=1.834, 95%CI: 1.107-3.039, Pz=0.019). No significant association was established for the HLA-DP following alleles: DPB1⁎02:01, DPB1⁎02:02, DPB1⁎04:01, DPB1⁎05:01, rs4282438, and rs3077.* Conclusion. *HLA-DP gene polymorphisms (HLA-DPB1⁎03:01, DPB1⁎04:02, DPB1⁎13:01, rs9277535, and rs3117027) were significantly associated with cervical cancer.

## 1. Introduction

Cervical cancer is the second most commonly diagnosed cancer and third leading cause of cancer-related mortality among women in less developed countries [1]. In only one year, 2012, there were an estimated 527,600 new cervical cancer cases and 265,700 deaths worldwide [2]. Human papillomavirus (HPV) has been widely accepted as a risk factor of cervical carcinogenesis [3]; however, HPV infections have developed to persistent infection in only a very few women and to cervical cancer in an even smaller proportion [4]. Many infected women have spontaneously HPV clear through immune response [5], suggesting that other factors such as host factors may contribute to the progression of the disease[6]. Understanding the relationship between host factors and cervical cancer risk is necessary to comprehend the striking heterogeneity in anti-HPV and provide evidence for rational design of host-directed therapy.

Human leukocyte antigens (HLA) Class II genes, which encoded by DR, DQ, and DP genes, are mainly expressed in antigen presentation cells. They are essential for the presentation of viral peptides to the immune system, including HPV [18, 19]. HLA II genes are highly polymorphic, and genetic variability of the HLA II alleles may lead to variations of the antigen-recognition of antigen presentation cells, thus resulting in the body being susceptible or resistant to HPV infection and consequentially affecting the results of the infection [19–21]. So far, more than 200 articles focusing on the relationship of HLA and cervical cancer have been published in the past 10 years; meanwhile many researches have investigated the relationship of HLA gene polymorphisms and cervical cancer risk [22, 23]. More importantly, HLA-DQA1, DQB1, and DRB1 gene polymorphisms have been found associated with the risk of cervical cancer in meta-analysis [24–26].

Recently, a number of genome-wide association studies (GWAS) have been performed to investigate the association between specific HLA II alleles and cervical cancer in Asia and Europe [7, 11, 13, 15], and many case-control studies have reported the association of HLA-DPA1, DPB1, and DPB2 with cervical cancer. Based on these studies, many alleles like HLA-DPB1*∗*02:02, DPB1*∗*03:01, DPB1*∗*04:02, DPB1*∗*05:01, DPB1*∗*13:01, rs9277535 (DPB1), rs4282438 (DPB2), rs3117027 (DPB2), and rs3077 (DPA1) were reported to be significantly associated with cervical cancer [8–10, 12, 14, 16, 17]. However, the results are inconclusive and controversial. For example, only in four GWAS focusing on investigation of susceptibility loci for cervical cancer, the results of associations between HLA-DPB1*∗*02:01, DPB1*∗*02:02, DPB1*∗*03:01, DPB1*∗*04:01, DPB1*∗*04:02, DPB1*∗*05:01, and DPB1*∗*13:01 and cervical cancer risk were not consistent [7, 11, 13, 15]. Since a single-center study may have an inadequate sample size and lack statistical power to obtain reliable conclusions and no previous meta-analyses in the literature had covered this research question, we conducted a meta-analysis of all eligible studies including the four GWAS to obtain a more precise estimation of the associations.

## 2. Methods

### 2.1. Search Strategy

To identify eligible studies, we systematically searched PubMed, EMBASE, ScienceDirect, Web of Science, Chinese National Knowledge Infrastructure (CNKI), and Wanfang databases. The keywords used for search were as follows: ‘‘cervical cancer” or ‘‘cervical carcinoma” or ‘‘uterine cervical carcinoma” and ‘‘HLA-DP” or ‘‘human leukocyte antigen” or ‘‘HLA antigen”. There were no limitations on language and publication year. The last search was updated on July 30, 2017. We also retrieved the references of all relevant articles to identify additional eligible studies.

### 2.2. Inclusion and Exclusion Criteria

Eligible studies must meet the following inclusion criteria: (a) case-control studies; (b) evaluating the association between HLA-DP gene polymorphisms (DPB1*∗*02:01, DPB1*∗*02:02, DPB1*∗*03:01, DPB1*∗*04:01, DPB1*∗*04:02, DPB1*∗*05:01, DPB1*∗*13:01, rs4282438, rs9277535, rs3077, and rs3117027) and cervical cancer (including cervical cancer in situ) risk; (c) available genotype frequencies; and (d) the genotype distribution in control groups being in the Hardy-Weinberg equilibrium (HWE).

Exclusion criteria of studies were as follows: (a) letters, reviews, and case reports; (b) lack of genotype frequency data; and (c) duplicate publication. In addition, if multiple studies had overlapping data, only those with complete data were included.

### 2.3. Data Extraction

Two authors independently selected the relevant articles and extracted the following data: first author's name, publication year, country, genotyping methods, number of cases and controls, genotype and allele frequency, and evidence of HWE in controls. Any controversy was resolved by discussion between the authors.

### 2.4. Statistical Analysis

HWE in the control group of each study was examined by goodness-of-fit chi-square test, and P HWE< 0.05 was considered as a deviation from HWE. The association between HLA-DP gene polymorphisms (DPB1*∗*02:01, DPB1*∗*02:02, DPB1*∗*03:01, DPB1*∗*04:01, DPB1*∗*04:02, DPB1*∗*05:01, DPB1*∗*13:01, rs4282438, rs9277535, rs3077, and rs3117027) and cervical cancer risk was evaluated by pooled OR and 95% CI. The Z test was used to investigate the significance of the pooled OR, and P_Z_<0.05 was considered as statistically significant. The heterogeneity between studies was assessed by the chi-square-based Q-test and I^2^ tests. If the P < 0.05 or I^2^>50%, indicating the existence of between-study heterogeneity, then the random-effects model was used to calculate the pooled ORs; otherwise, the fixed-effects model was applied to the analysis. The stability of the result was evaluated by sensitivity analysis. Egger's test and Begg's test were used to determine the publication bias among studies, and P_E_<0.05 was considered significant. All statistical tests were performed with the STATA software (version 12.0; StataCorp, College Station, TX, USA).

## 3. Results

### 3.1. Study Selection and Characteristics

**Figure 1:**
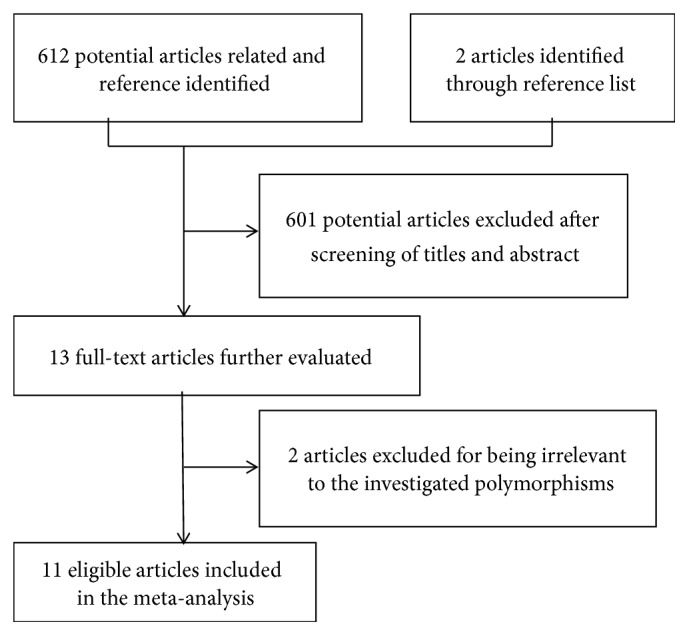
Flow chart of study selection in the meta-analysis.

The study selection process is shown in Figure 1. A total of 614 articles were initially retrieved from electronic databases including PubMed, EMBASE, ScienceDirect, Web of Science, CNKI, and Wanfang databases. After reviewing the titles, abstracts, and full text, we excluded 603 irrelevant studies. Finally, 11 articles published between 2007 and 2016 assessing the association between HLA-DP gene polymorphism and cervical cancer risk were included in the current meta-analysis. The main characteristics of all eligible studies are shown in Table 1. All the included studies were conducted in Europe or Asia. For HLA-DPB1*∗*02:01, DPB1*∗*03:01, and DPB1*∗*04:01, there were six articles included. For HLA-DPB1*∗*05:01 and DPB1*∗*13:01, there were five articles included. For HLA-DPB1*∗*02:02, DPB1*∗*04:02, rs4282438, and rs9277535, there were four articles included. For HLA-DP rs3077 and rs3117027, there were three articles included. For different allele, the number of cases ranged from 882 to 5008, and the number of controls ranged from 1150 to 9322 (Table 2).

### 3.2. Quantitative Data Synthesis

**Figure 2:**
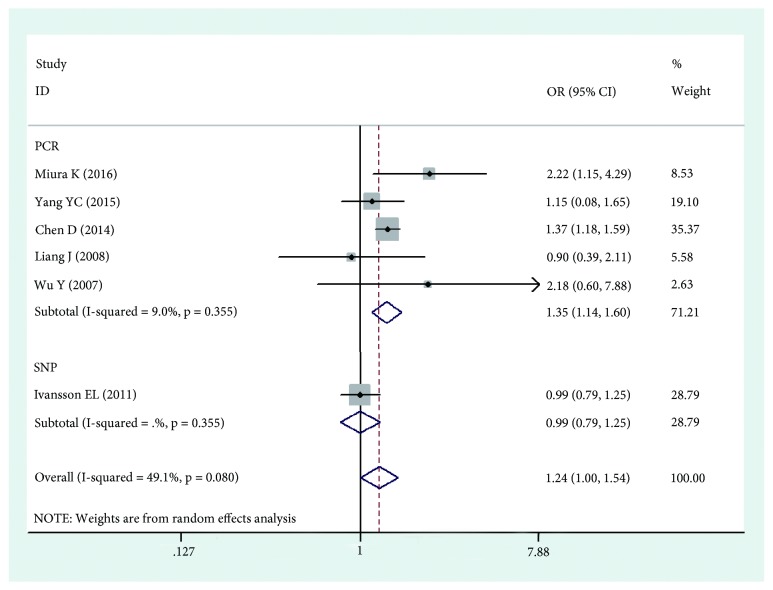
Forest plot of effect estimates for DPB1*∗*03:01 polymorphism and cervical cancer risk.

**Figure 3:**
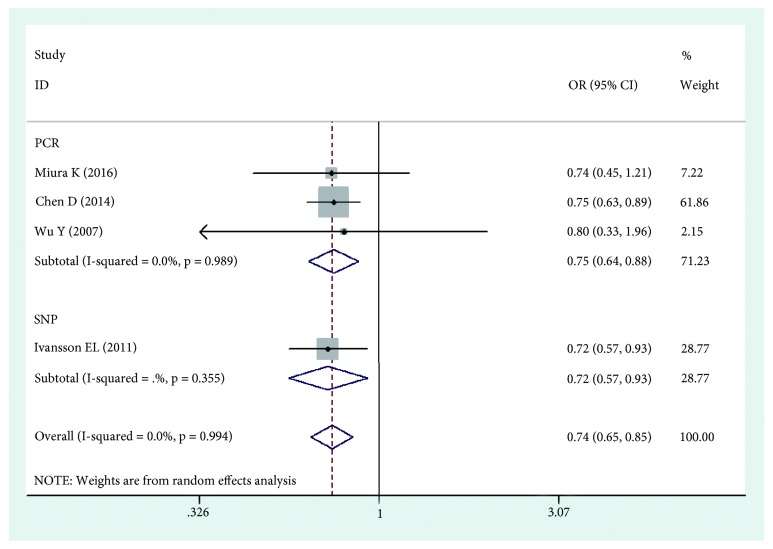
Forest plot of effect estimates for DPB1*∗*04:02 polymorphism and cervical cancer risk.

**Figure 4:**
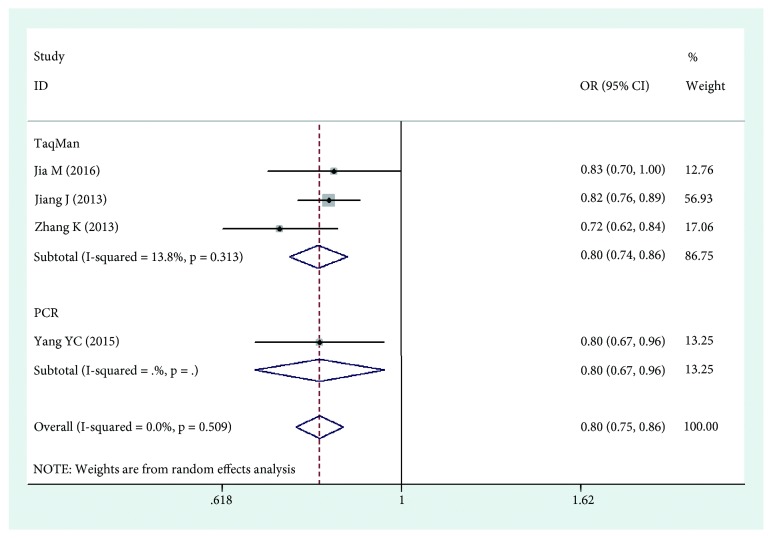
Forest plot of effect estimates for rs9277535 G allele and cervical cancer risk.

The results of this meta-analysis are shown in Tables 2, 3, and 4. Based on the results, we found that HLA-DPB1*∗*03:01 was significantly associated with an increased risk of cervical cancer (OR=1.252, 95%CI: 1.116-1.403, Pz=0.001) (Figure 2), while HLA-DPB1*∗*04:02 and HLA-DP rs3117027 G allele were significantly associated with a decreased risk of cervical cancer risk (OR=0.744, 95%CI: 0.652-0.848, Pz=0.001; OR=0.790, 95%CI: 0.745-0.837, Pz=0.001) (Figure 3), and HLA-DP rs9277535 G allele was significantly associated with a decreased risk of cervical cancer risk in Asia (OR=0.802, 95%CI: 0.753-0.855, Pz=0.001) (Figure 4). Though not significant, HLA-DPB1*∗*13:01 showed a tendency of association with an increased risk of cervical cancer in Europe and Asia (OR=1.518, 95%CI: 0.954-2.416, Pz=0.078).

**Figure 5:**
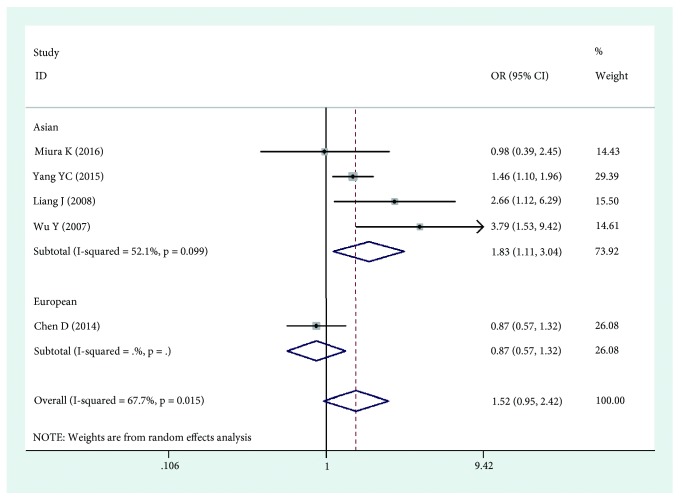
Forest plot of effect estimates for DPB1*∗*13:01 polymorphism and cervical cancer risk.

In order to clarify interactions between HLA-DPB1*∗*02:01, DPB1*∗*03:01, DPB1*∗*04:01, DPB1*∗*05:01, and DPB1*∗*13:01 and cervical cancer in Asia, we conducted subgroup analyses based on race system, and we found that HLA-DPB1*∗*13:01 was significantly associated with an increased risk of cervical cancer in Asia (OR=1.834, 95%CI: 1.107-3.039, Pz=0.019) (Figure 5), and though not significant, HLA-DPB1*∗*03:01 showed a tendency of association with an increased risk of cervical cancer in Asia (OR=1.317, 95%CI: 0.987-1.757, Pz=0.061).

The different typing methods used in different laboratories might lead to heterogeneity, so we conducted subgroup analyses based on genotyping method. The results showed that HLA-DPB1*∗*03:01 was significantly associated with an increased risk of cervical cancer (OR=1.354, 95%CI: 1.144-1.604, Pz=0.001) (Figure 2), while HLA-DPB1*∗*04:02 was significantly associated with a decreased risk of cervical cancer risk (OR=0.750, 95%CI: 0.642-0.877, Pz=0.001) (Figure 3), and HLA-DP rs9277535 G allele was significantly associated with a decreased risk of cervical cancer risk in Asia (OR=0.800, 95%CI: 0.740-0.865, Pz=0.001) (Figure 4).

We also found that the following HLA-DP alleles: DPB1*∗*02:01, DPB1*∗*02:02, DPB1*∗*04:01, DPB1*∗*05:01, rs4282438, and rs3077 were not significantly associated with cervical cancer risk.

### 3.3. Sensitivity Analysis and Publication Bias

**Figure 6:**
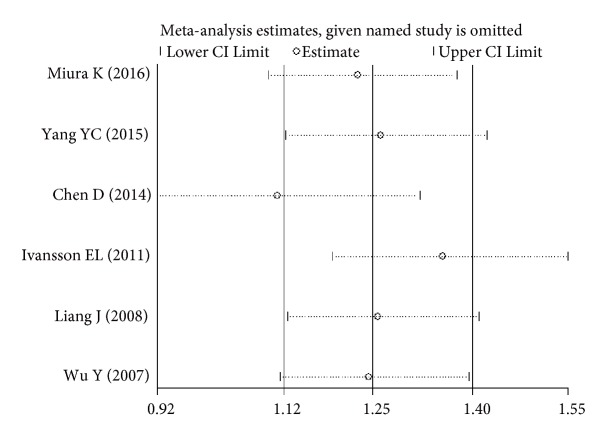
Sensitivity analysis of the pooled ORs and 95%CIs for DPB1*∗*03:01 polymorphism.

**Figure 7:**
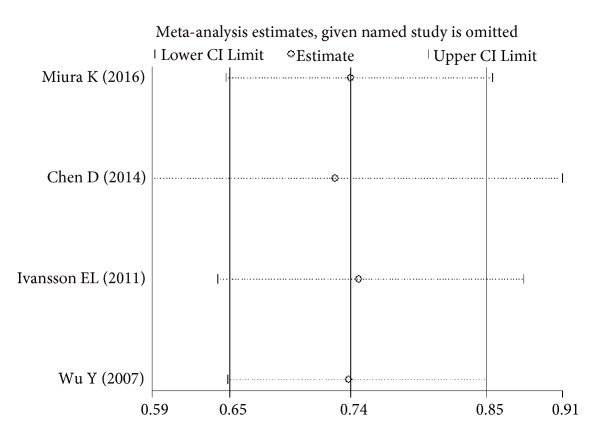
Sensitivity analysis of the pooled ORs and 95%CIs for DPB1*∗*04:02 polymorphism.

**Figure 8:**
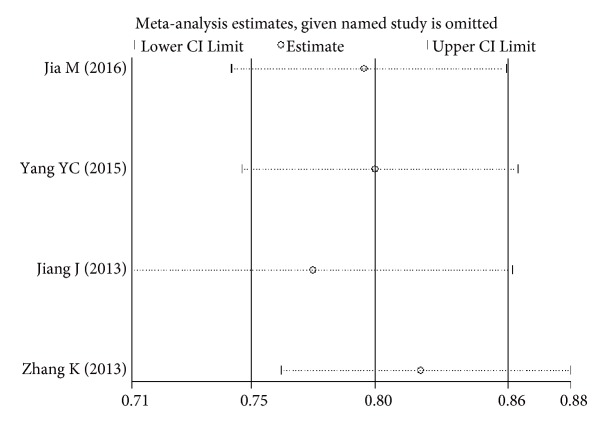
Sensitivity analysis of the pooled ORs and 95%CIs for rs9277535 G allele.

**Figure 9:**
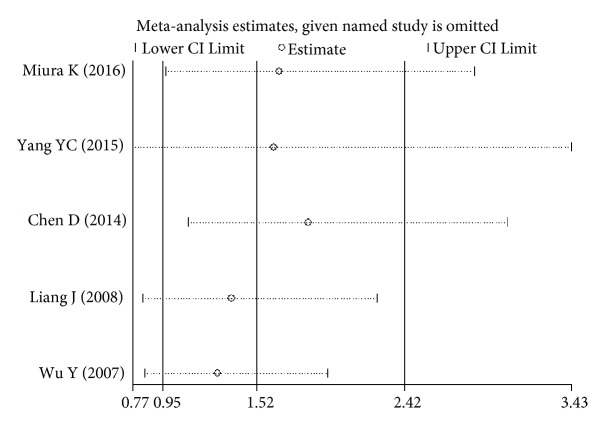
Sensitivity analysis of the pooled ORs and 95%CIs for DPB1*∗*13:01 polymorphism.

The sensitivity analysis showed that no single study altered the pooled ORs qualitatively, which provided the evidence of the stability of the meta-analysis (Figures 6–9). As shown in Tables 2, 3, and 4, there was no publication bias for any of the alleles.

## 4. Discussion

Individuals with HPV infection may have distinct results, for example, natural virus clearance, persistent infection with no symptom, or development of premalignant lesion or invasive cancer, even with the same HPV exposure [27–29]. HLA gene polymorphisms, including HLA-DP gene polymorphisms, might be at least one of the reasons for these differences. However, recent studies investigating the association between HLA-DP gene polymorphisms and cervical cancer have been inconsistent. For example, in two GWAS focusing on the investigation of susceptibility loci for cervical cancer in Europe, Chen D reported that HLA-DPB1*∗*04:02 and rs3117027 G allele were significantly associated with a decreased risk of cervical cancer risk, while HLA-DPB1*∗*03:01 was significantly associated with an increased risk of cervical cancer risk, and HLA-DPB1*∗*02:01, DPB1*∗*04:01, DPB1*∗*13:01, and rs4282438 were not associated with cervical cancer risk [10, 11]. However, in another GWAS carried by Ivansson EL, HLA-DPB1*∗*0201 and DPB1*∗*0402 showed protective effect, and HLA-DPB1*∗*0301, DPB1*∗*0401, and DPB1*∗*0501 showed no significant effect on cervical cancer risk [15]. Furthermore, a GWAS carried out in Chinese showed that HLA-DPB1*∗*03:01 and DPB1*∗*04:01 were associated with susceptibility to cervical cancer, while HLA-DPB1*∗*05:01 and rs4282438 G allele showed protective effects[13]. In addition, in a GWAS investigating susceptibility loci for cervical cancer in Japanese population, HLA-DPB1*∗*02:01, DPB1*∗*02:02, DPB1*∗*03:01, DPB1*∗*04:01, DPB1*∗*04:02, DPB1*∗*05:01, DPB1*∗*13:01, and rs4282438 were not significantly associated with cervical cancer [7]. On the other hand, the associations between HLA-DP rs977535, rs3117027, and rs3077 and cervical cancer risk were inconsistent in Chinese population [8–12, 14].

Meta-analysis is a powerful tool to gather data from individual studies and thus enhance the statistical power of the analysis and reduce random error of false-positive or false-negative associations to obtain reliable results [30]. In the present meta-analysis, a total of 11 studies, including 11 HLA-DP alleles, with at most 5008 cervical cancer cases and 9322 healthy controls, were evaluated. Based on the results, we found that HLA-DPB1*∗*04:02 and rs3117027 G allele were strongly related to cervical cancer as protective factors (95%CI: 0.652-0.848, Pz=0.001; 95%CI: 0.745-0.837, Pz=0.001), while HLA-DPB1*∗*03:01 might be regarded as risk factors (95%CI: 1.116-1.403, Pz=0.001), which was consistent with the GWAS carried out in China and Europe [12, 13, 15]. In addition, HLA-DP rs9277535 G allele and DPB1*∗*13:01 might be regarded as protective factor (95%CI: 0.753-0.855, Pz=0.001) and risk factor (95%CI: 1.107-3.039, Pz=0.019) of cervical cancer in Asia, respectively, while HLA-DPB1*∗*13:01 was not regarded as risk factor for cervical cancer in the GWAS of cervical cancer in Japanese [7]; that might be related to the relatively small number of cases of the GWAS, thus having low weight. However, HLA-DPB1*∗*02:01, DPB1*∗*02:02, DPB1*∗*04:01, DPB1*∗*05:01, rs4282438, and rs3077 did not show significant associations with cervical cancer in our meta-analysis. In two GWAS, HLA-DPB1*∗*02:01, DPB1*∗*04:01, DPB1*∗*0501, and rs4282438 showed no significant effect on cervical cancer risk [10, 15]. Furthermore, in another GWAS investigating susceptibility loci for cervical cancer in Japanese population, HLA-DPB1*∗*02:01, DPB1*∗*02:02, DPB1*∗*04:01, and rs4282438 were not significantly associated with cervical cancer [7]. All provided the evidence of the reliability of the meta-analysis. Additionally, we found that HLA-DPB1*∗*02:01, DPB1*∗*02:02, DPB1*∗*05:01, DPB1*∗*13:01, rs4282438, and rs3077 in all groups had high heterogeneity, but there was no publication bias.

In order to clarify interactions between HLA-DP gene polymorphisms and cervical cancer in Asia, we conducted a subgroup analysis. The results showed that HLA-DPB1*∗*13:01 was significantly associated with cervical cancer, and HLA-DPB1*∗*03:01 showed a tendency of association with an increased risk of cervical cancer in Asia, while HLA-DPB1*∗*02:01, DPB1*∗*04:01, and DPB1*∗*05:01 were not associated with cervical cancer in Asia either, which implies that some alleles have the same effect in Asia and Europe. The results of subgroup analysis of seven alleles based on genotyping method also showed that HLA-DPB1*∗*03:01 was associated with an increased risk of cervical cancer, and HLA-DPB1*∗*04:02 and rs9277535 G allele were associated with a decreased risk of cervical cancer, which implicated that genotyping method may not affect the results.

Although the correlation of cervical cancer with HLA-DP genes has been demonstrated by various studies, the molecular mechanisms underlying the association are still not elucidated. Tumor development is preceded by chronic inflammation and immune responses, whether towards the infectious agent itself or against tumor antigens. Human tumor cells express diverse types of antigens, depending on the etiology and pathogenesis of the disease. HLA-DP alleles may affect the way the human body involved in the immune system and in cell cycle. Some alleles are considered protective while others increase the risk of developing a certain condition. On the basis of our results, it is reasonable to infer that HLA-DPB1*∗*03:01, DPB1*∗*13:01, DPB1*∗*04:02, and rs9277535 alleles are able to affect the antigen presentation and cellular expression of HLA-DP molecules. These specific HLA-DP alleles are therefore likely to associate with persistent HPV infections and thus increase or decrease the risk of cervical cancer.

Some limitations existed in the present meta-analysis: First, not all alleles were reported in each study. For example, in a GWAS investigating susceptibility loci for cervical cancer in Han Chinese, the associations of HLA-DPB1*∗*03:01, DPB1*∗*04:01, DPB1*∗*05:01, and DPB1*∗*13:01 with cancer risk were also investigated [13], but we did not include the four alleles in the meta-analysis, because we could not obtain the precise allele frequency. Second, our results were not adjusted. Since age, ethnicity, family history, environmental factors, and HPV infection type are important factors for development of cervical cancer, it is better to conduct the precise analysis adjusted by the above varieties. However, not all studies included have reported age, family history, and the situation of HPV infection, etc. Approximately 200 HPV types have been identified to date, and HPV types are associated with the malignant of disease; for example, HPV types 16 and 18 are responsible for approximately 60%–80% of all cervical cancer cases, while types 31 and 52 account for the majority of the remaining cases [7, 31, 32]. Third, not all alleles were included in the current meta-analysis, such as rs3117027 [8, 10, 11] and rs4282438of HLA-DPB2 [7, 13]. Finally, some studies included in the meta-analysis took cervical intraepithelial neoplasia III (CINIII) females as cases into analysis. Considering that CINIII is the important stage for developing cervical cancer, we also enrolled it.

## 5. Conclusion

To the best of our knowledge, no previous meta-analysis has comprehensively assessed the associations between the eleven alleles and cervical cancer risk. Since the number of cases and controls included in the current meta-analysis is relative huge, our results would be relatively reliable, and we could conclude that HLA-DP gene polymorphisms (DPB1*∗*03:01, DPB1*∗*04:02, DPB1*∗*13:01, rs9277535, and rs3117027) were significantly associated with cervical cancer, which would be regarded as early warning factors. More well-designed large-scale studies including individuals from various countries and regions are still needed to determine the associations between HLA-DP gene polymorphisms and the risk of cervical cancer.

## Figures and Tables

**Table 1:** Main characteristics of all studies included in the meta-analysis.

First author	Year	Country	Case	Control	Genotyping method	Polymorphisms
Miura K [7]	2016	Japan	214	288	PCR-SBT	DPB1*∗*02:01, DPB1*∗*02:02, DPB1*∗*03:01, DPB1*∗*04:01, DPB1*∗*04:02, DPB1*∗*05:01, DPB1*∗*13:01, rs4282438

Jia M [8]	2016	China	593	4074	TaqMan assay	rs4282438, rs9277535, rs3117027, rs3077

Yang YC [9]	2015	Taiwan	473	676	PCR, sequencing	DPB1*∗*02:01, DPB1*∗*02:02, DPB1*∗*03:01, DPB1*∗*04:01, DPB1*∗*05:01, DPB1*∗*13:01, rs9277535

Chen D [10]	2014	Sweden	1034	3948	PCR, sequencing	DPB1*∗*02:01, DPB1*∗*03:01, DPB1*∗*04:01, DPB1*∗*04:02, DPB1*∗*13:01, rs4282438, rs3117027

Chen D [11]	2013	Sweden	2215	5072	GENios Pro platform	rs3117027

Jiang J [12]	2013	China	2317	2109	TaqMan assay	rs9277535, rs3077

Shi Y [13]	2013	China	5531	10224	Affymetrix Axiom Genome-Wide CHB1 Array	rs4282438

Zhang K [14]	2013	China	831	573	TaqMan assay	rs9277535, rs3077

Ivansson EL [15]	2011	Sweden	1076	1426	Affymetrix Genome-Wide Human SNP Array	DPB1*∗*02:01, DPB1*∗*03:01, DPB1*∗*04:01, DPB1*∗*04:02, DPB1*∗*05:01

Liang J [16]	2008	China	126	88	PCR-SBT	DPB1*∗*02:0102, DPB1*∗*02:02, DPB1*∗*03:0101, DPB1*∗*04:0101, DPB1*∗*05:01, DPB1*∗*13:01

Wu Y [17]	2007	China	133	98	PCR-SBT	DPB1*∗*02:0102, DPB1*∗*02:02, DPB1*∗*03:0101, DPB1*∗*04:0101, DPB1*∗*04:02, DPB1*∗*05:01, DPB1*∗*13:01

PCR-SBT: polymerase chain reaction sequence-based typing; SNP: single nucleotide polymorphisms.

**Table 2:** Meta-analysis of associations between HLA-DP alleles and cervical cancer.

Alleles	No. of studies	Case (2n)	Control (2n)	Heterogeneity P value	I^2^ value (%) for heterogeneity test	Model	OR (95%CI)	P value	Z	P value for Egger's (Begg's) bias test
DPB1*∗*02:01	6	743/5916	1397/10888	0.006	69.1	R	0.915 (0.741-1.130)	0.409	0.82	0.981 (1.000)

DPB1*∗*02:02	4	103/1764	132/2300	0.071	57.4	R	1.261 (0.740-2.418)	0.394	0.85	0.107 (0.089)

DPB1*∗*03:01	6	652/5878	1032/10888	0.080	49.1	F	1.252 (1.116-1.403)	0.001	3.84	0.853 (1.000)

DPB1*∗*04:01	6	2015/5892	4215/10878	0.210	30.1	F	1.009 (0.932-1.092)	0.821	0.23	0.240 (0.133)

DPB1*∗*04:02	4	412/4734	1075/9372	0.994	0.0	F	0.744 (0.652-0.848)	0.001	4.41	0.781 (1.000)

DPB1*∗*05:01	5	744/3916	1031/3248	0.007	71.4	R	0.953 (0.728-1.248)	0.727	0.35	0.129 (0.086)

DPB1*∗*13:01	5	177/3840	244/10166	0.015	67.7	R	1.518 (0.954-2.416)	0.078	1.76	0.531 (0.806)

rs4282438-T allele	4	5309/10016	12197/18038	0.001	90.3	R	0.914 (0.688-1.213)	0.533	0.62	0.382 (0.734)

rs9277535 G allele*∗*	4	4268/8266	4297/7402	0.509	0.0	F	0.802 (0.753-0.855)	0.001	6.76	0.602 (1.000)

rs3117027-G allele	3	4994/7588	13159/18644	0.759	0.0	F	0.790 (0.745-0.837)	0.001	7.92	0.055 (0.296)

rs3077-C allele*∗*	3	3494/5613	3374/5022	0.001	94.7	R	0.816 (0.560-1.189)	0.290	1.06	0.957 (1.000)

The HLA-DP rs9277535 and rs3077 were just studied among Asian population.

**Table 3:** Meta-analysis of associations between HLA-DP alleles and cervical cancer in Asian.

Alleles	No. of studies	Case (2n)	Control (2n)	Heterogeneity P value	I^2^ value (%) for heterogeneity test	Model	OR (95%CI)	P value	Z	P value for Egger's (Begg's) bias test
DPB1*∗*02:01	4	310/1764	369/2300	0.088	54.1	R	1.031 (0.781-1.362)	0.829	0.22	0.109 (0.308)

DPB1*∗*03:01	4	98/1764	98/2300	0.228	30.7	F	1.317 (0.987-1.757)	0.061	1.87	0.570 (0.734)

DPB1*∗*04:01	4	112/1764	158/2300	0.080	55.7	R	0.829 (0.524-1.314)	0.426	0.80	0.277 (0.308)

DPB1*∗*05:01	4	679/1764	1012/2300	0.034	65.4	R	0.874 (0.680-1.123)	0.291	1.06	0.358 (0.308)

DPB1*∗*13:01	4	150/1764	126/2300	0.099	52.1	R	1.834 (1.107-3.039)	0.019	2.36	0.495 (0.734)

**Table 4:** Subgroup meta-analysis of associations between HLA-DP alleles and cervical cancer based on genotyping method.

Alleles	No. of studies	Genotyping method	Case (2n)	Control (2n)	Heterogeneity P value	I^2^ value (%) for heterogeneity test	Model	OR (95%CI)	P value	Z	P value for Egger's (Begg's) bias test
DPB1*∗*02:01	5	PCR	506/3764	1263/9940	0.011	69.2	R	0.960 (0.749-1.231)	0.750	-0.24	0.996 (1.000)

DPB1*∗*03:01	5	PCR	372/3726	908/9940	0.355	9.0	F	1.354 (1.144-1.604)	0.001	0.24	0.804 (0.806)

DPB1*∗*04:01	5	PCR	1068/3740	3798/9930	0.129	43.9	F	0.951 (0.748-1.210)	0.685	1.71	0.319 (0.086)

DPB1*∗*04:02	3	PCR	218/2582	961/8424	0.989	0.0	F	0.750 (0.642-0.877)	0.001	1.02	0.640 (1.000)

DPB1*∗*05:01	4	PCR	679/1764	1012/2300	0.034	65.4	R	0.874 (0.680-1.123)	0.291	0.00	0.358 (0.308)

rs4282438	2	PCR	2244/2496	8012/8470	0.159	49.6	F	0.842 (0.653-1.087)	0.186	0.00	- (1.000)

Rs9277535	3	TaqMan	3666/7320	3370/6050	0.313	13.8	F	0.800 (0.740-0.865)	0.001	0.00	0.695 (1.000)
